# Oxidative Stress in Aging Human Skin

**DOI:** 10.3390/biom5020545

**Published:** 2015-04-21

**Authors:** Mark Rinnerthaler, Johannes Bischof, Maria Karolin Streubel, Andrea Trost, Klaus Richter

**Affiliations:** 1 Department of Cell Biology, Division of Genetics, University of Salzburg, Salzburg 5020, Austria; E-Mails: mark.rinnerthaler@sbg.ac.at (M.R.); johannes.bischof@stud.sbg.ac.at (J.B.); mariakarolin.streubel@stud.sbg.ac.at (M.K.S.); 2 Department of Ophthalmology and Optometry, Paracelsus Medical University, Muellner Hauptstrasse 48, 5020 Salzburg, Austria; E-Mail: A.Zurl@salk.at

**Keywords:** lipofuscin, AGEs, cornified envelope, calcium gradient, extrinsic and intrinsic aging, DNA damage, lipid peroxidation, antioxidants, carbonylation

## Abstract

Oxidative stress in skin plays a major role in the aging process. This is true for intrinsic aging and even more for extrinsic aging. Although the results are quite different in dermis and epidermis, extrinsic aging is driven to a large extent by oxidative stress caused by UV irradiation. In this review the overall effects of oxidative stress are discussed as well as the sources of ROS including the mitochondrial ETC, peroxisomal and ER localized proteins, the Fenton reaction, and such enzymes as cyclooxygenases, lipoxygenases, xanthine oxidases, and NADPH oxidases. Furthermore, the defense mechanisms against oxidative stress ranging from enzymes like superoxide dismutases, catalases, peroxiredoxins, and GSH peroxidases to organic compounds such as L-ascorbate, α-tocopherol, beta-carotene, uric acid, CoQ10, and glutathione are described in more detail. In addition the oxidative stress induced modifications caused to proteins, lipids and DNA are discussed. Finally age-related changes of the skin are also a topic of this review. They include a disruption of the epidermal calcium gradient in old skin with an accompanying change in the composition of the cornified envelope. This modified cornified envelope also leads to an altered anti-oxidative capacity and a reduced barrier function of the epidermis.

## 1. Introduction: The Skin as a Model for the “ROS-Aging” Connection

Aging research has focused on a central finding that dates back to the year 1956. In this year Denham Harman proposed that reactive oxygen species (ROS) accumulate over time and are a main contributor to the aging process [1]. This concept was broadened 16 years later by Harman himself by identifying mitochondria as the main source of ROS, forming the basis for the mitochondrial free radical theory of aging [2]. Especially in the last decade, serious doubts arose that ROS are in fact the most important components that are fueling aging [3]. For example it was shown that SOD^+/−^ mice had a clear increase in the ROS load but a quite normal lifespan [4]. Even the opposite was observed. In several cases it could be demonstrated that an increase in oxidative stress led to an increase in lifespan (summarized in more detail by Ristow and Schmeisser in [5]). However, if the free radical theory of aging holds true in any organ of the human body it is in the skin. Not only is the ROS load in this organ higher than in any other organ, but in many cases a clear correlation between the ROS originating from external and internal insults and a pro-aging effect can be found. A characteristic of this organ is also the fact that extrinsic aging is at least as important as intrinsic aging.

Intrinsic aging is described as a result of genetic factors and corporal changes that occur/appear during the normal aging process, whereas extrinsic aging focuses on aging process accelerated by environmental influences [6]. It was proposed that only three percent of all aging factors have a genetic background [7]. Aging leads to a thinning of epidermal as well as dermal skin layers. The skin also loses sensibility due to decreased production of sex hormones and a decreased number of nerve-endings. In addition the skin gets dryer and gradually loses the function to serve as a first barrier against the environment [8,9,10]. In contrast, focusing on extrinsic aging, it seems that the skin gets thicker and completely changes its composition [10,11]. Extrinsic aging is synonymous with photoaging as UV-radiation has severe consequences for exposed skin. However, there are many more environmental factors influencing skin aging, as discussed later [12].

## 2. The Aging Process in the Dermis

Human skin essentially consists of two layers: The epidermis on the outside and the dermis below, both are attached to each other via the basal lamina. Aging has a quite different appearance depending if either dermis or epidermis is considered. In the dermis the disruption of the extracellular matrix plays the most obvious role which is true for intrinsic as well as extrinsic aging. The results are fine wrinkles due to the reduction of collagen, elastic fibers, and hyaluronic acid.

In the context of intrinsic as well as extrinsic aging the disruption of the extracellular matrix plays an important role. Enzymes in the extracellular matrix (ECM) are responsible for the processing of elastic fibers, collagens, and proteoglycans [13,14]. Elastic fibers are structures of fibrillin-rich microfibrils, glycoproteins, elastins, and other different proteins [15]. Fibrillins and fibrillary collagens are glycoproteins with modifications of different branched oligosaccharides whereas proteoglycans consist of one long, unbranched glycosaminoglycan side chain. These glycoproteins are connected to each other with hyaluronic acid and build up a dermal network [16]. Long collagen fibrils from collagen I and III are interwoven and form an intra-dermal net which is anchored to the dermal-epidermal junction by collagen VII [16,17,18].

In the course of intrinsic aging collagen and elastic fibers stay intact but are further apart forming a wider-mashed network [16]. During extrinsic aging the skin dramatically loses collagen I, III and VII [19,20]. The long collagen fibrils, elastic fibers, glycoproteins and glycosaminoglycans are no longer interwoven to form a functional network but form an unorganised dermal-spreaded agglomeration [16]. This disruption is further aggravated by elastases produced by neutrophils that migrate to the dermis after inflammation or UV exposure [21] and by the activation of matrix metalloproteases (MMPs). Especially MMP1, 2, 3 and 9 are heavily involved in the degradation of the dermal extracellular matrix [22]. Collagen can only be cleaved by MMP1 and later on completely degraded by MMPs 2, 3 and 9. MMPs 2 and 9 are also able to degrade elastic fibers [23,24]. During the aging process these MMPs are upregulated while their inhibitors, namely TIMP1 and 3, are downregulated [24,25].

During photoaging this degradation is significantly accelerated by a process called ECM turnover [26]. UV-irradiation, especially UVA and UVB results in the production of ROS as well as the activation of cell surface receptors [27] leading to an activation of MAP-kinase p38, JNK (c-Jun amino-terminal kinase) and ERK (extracellular signal-regulated kinase) and the recruitment of c-Fos and c-Jun. This leads to the expression of the transcription factor activator protein 1 (AP-1) resulting in the expression of MMP1, 3 and 9 [28] in fibroblasts and keratinocytes [26]. AP-1 also inhibits TGF-β which is responsible for collagen production [29]. The AP-1 mediated MMP expression leads to an increased degradation of the ECM. This process is reinforced by the production of ROS also resulting in the activation of MAP kinases and in addition leading to the expression of NF-κB.

Interestingly NF-κB and AP-1 are also important for the balance between proliferation and apoptosis [30]. An imbalance of these two processes, especially in context with proto-oncogenes like c-Fos [31] may be heavily involved in carcinogenesis in aged human skin [32,33]. This is especially relevant for senescent skin cells [24].

In addition to aging it is well known that UV-irradiation induces mutations in the tumor suppressor p53 leading to actinic keratosis in a first step, that can further develop to skin cancers [34]. UV-irradiation of skin also leads to IGF-1 expression and secretion in dermal fibroblasts, which in turn stimulate the epidermal IGF-1 receptor (IGF-1R) in keratinocytes. Upon IGF-1R activation, keratinocytes get more resistant to UVB-irradiation, and do not undergo apoptosis, but stop the cell cycle with the side effect of becoming senescent. The loss of IGF-1 in aged skin leads to a higher apoptotic rate, but the surviving cells do not become senescent, which contributes together with oxidative stress to an increase in the formation of squamous cell carcinomas and basal cell epitheliomas in aged skin [35,36,37,38]. Another age-associated skin disease is vitiligo-similar appearance. This disease is a result of damaged melanocytes leading to white spots (idiopathic guttatehypomelanosis) [39] as well as lighter-tanned skin [40,41], visible only in aged patients. This hypopigmentation is a result of a ROS-imbalance in the skin [30,42] leading to an impairment of either the differentiation and survival of melanocytes or the melanin transport to keratinocytes, especially in aged and photoaged skin [38,40,41,42].

## 3. The Epidermis: The Process of Cornification and Aging

### 3.1. The Cornified Envelope Formation

Histologically different layers in the epidermis can be distinguished. The *stratum basale* consists of the stem cells that are attached to the basal membrane that seperates the dermis from the epidermis. Following the *stratum basale*, the *stratum spinosum*, the *stratum lucidum* (in palms and soles), the *stratum granulosum*, *stratum corneum* and *stratum disjunctum* can be seen.

The process of cornification in human skin is a step-by-step process involving the crosslinking of various proteins. This extensive crosslinking leads to the formation of a multi-protein complex in the outermost layer of the epidermis. The first step of the cornification process takes place in a layer located above the *stratum basale*, the *stratum spinosum*. Three important proteins are expressed here: envoplakin [43], periplakin [44] and involucrin [45]. The two plakins form a complex with involucrin [46], creating a platform for subsequent crosslinking of further proteins [47]. The next step in the cornification process leads to the formation of lamellar bodies by the Golgi apparatus in the *stratum spinosum* and the *stratum granulosum* [48,49]. These granules are characterized by an enveloping lipid layer consisting of a multitude of lipids like glucosylceramides and sphingomyelins [50]. Within the lamellar bodies several enzymes like lipid processing enzymes, antimicrobial peptides, proteases and protease inhibitors as well as proteins like corneodesmosin, an adhesive protein, can be found [50,51,52,53,54]. After an influx of calcium into the keratinocytes [55] the lamellar bodies fuse with the plasma membrane. This leads to a replacement of phospholipid in the lipid bilayer with ω-OH-ceramides. These ceramides are further crosslinked with the periplakin-envoplakin-involucrin complex via transglutaminase 1 [56]. The calcium-dependent transglutaminase 1 [57] is responsible for the attachment of this complex to the lipid bilayer via Nε-(γ glutamyl) lysine (isopeptide) bonds [58]. The main component of the cornified envelope though is loricrin. Loricrin is highly expressed in the *stratum granulosum* [56] and is packed into granules directly after translation due to its high insolubility [59]. Transglutaminase 1 and 3 are responsible for the crosslinking of all loricrin proteins between each other and for crosslinking loricrin to a family of proteins called small proline rich repeat proteins (SPRRs). These proteins are very hydrophilic and help to increase the solubility of loricrin [60,61,62]. Our own published data indicates clearly that the addition of calcium to primary keratinocytes dramatically increases the expression of most of the SPRRs and loricrin. The loricrin-SPRR aggregate is then attached to the periplakin-envoplakin-involucrin scaffold at the cell membrane [56].

The cornification process proceeds by the attachment of various other proteins to the multi-protein complex at the cell membrane. One of these proteins is the calcium-regulated filaggrin [63]. Filaggrin is well known for bundling keratins into macrofibrils giving rise to the typical flattened shape of corneocytes [64]. The membrane-associated desmosomal keratins 1 and 10 begin to replace the pre-existing keratin 5 and 14 intermediate filament bundles (KIFs) aggregating them into tight bundles. This causes a significant change in the shape of the cells by changing cytoskeletal properties and cell-cell interactions [56]. Another calcium signal is then needed for the bundling of keratins into tonofilaments [65]. Other proteins attached to the cornified envelope complex are S100 protein family members. Some of the members of this protein family serve as substrates for transglutaminase 1 [61,66].

In a last step of cornification, the late cornified envelope proteins (LCE) are attached to the protein-lipid complexes [67]. Some of the members react and attach “group-wise” in response to external stimuli like calcium [68]. The resulting “cornified” cell now consists of a mega-protein-lipid skeleton and has degraded its nucleus, mitochondria and other organelles. Its ultimate fate is to build the barrier function of the skin and finally it is shed as a dead corneocyte [69].

### 3.2. The Calcium Dependence of the Cornified Envelope Formation

Calcium has an important role during the cornification process. It is not just an “on-off principle” since calcium regulates the expression of genes in a dose-dependent manner. Keratinocytes need low calcium concentrations for the renewal and division of stem cells and transit amplifying cells, whereas higher concentrations are needed for differentiation. To ensure the right concentration of calcium at the respective epidermal layer the epidermis has built up a calcium gradient. The low calcium concentration found in the *stratum basale* gradually increases through the s*tratum spinosum* until reaching its peak in the *stratum granulosum*. In the outermost layer, *the stratum corneum*, the calcium concentration sharply declines [70,71,72]. Basically most, if not all steps discussed above are strictly calcium dependent [73]. During the aging process the calcium gradient collapses and the composition of the cornified envelope changes drastically [72]. Our data show that epidermis obtained from young and middle-aged foreskin samples has a clear calcium peak in the *stratum granulosum*. Epidermis from old donors on the other hand has lost its peak and has an equal distribution of calcium in all epidermal layers [72]. Denda *et al.* were able to show the same rearrangement of calcium in epidermal layers of aged facial skin [74]. This leads to a changed composition of the cornified envelope: main components like loricrin and filaggrin are significantly down-regulated on the transcriptional and translational level while other components like envoplakin, periplakin, and involucrin show no transcriptional regulation in aged skin. The loss of loricrin and filaggrin seems to be compensated by increased levels of SPRRs since the expression of nearly all members of this family, with the exception of SPRR2G, are upregulated. This may represent a rescue mechanism to maintain the function and integrity of the cornified envelope. Transglutaminases, important for the cohesion of the protein-lipid complex, show constantly higher transcription levels in young skin than in old skin. Furthermore LCEs, some of them being substrates for transglutaminases, show either an up- or down-regulation during aging. Calcium-sensitive group 3 LCEs are upregulated during aging while group 2 LCEs are transcriptionally downregulated [68,72]. However, all S100 proteins are up-regulated during human skin aging [72]. Important in this context is the upregulation of S100A8 as it plays a role in the epidermal defense against oxidative stress. Besides this pronounced change in the calcium distribution and the resulting change in the composition of the cornified envelope, the transcription of many other genes [75] and such important regulators as miRNAs [76] are also altered during the aging process in the skin.

## 4. ROS Production in the Skin

**Figure 1:**
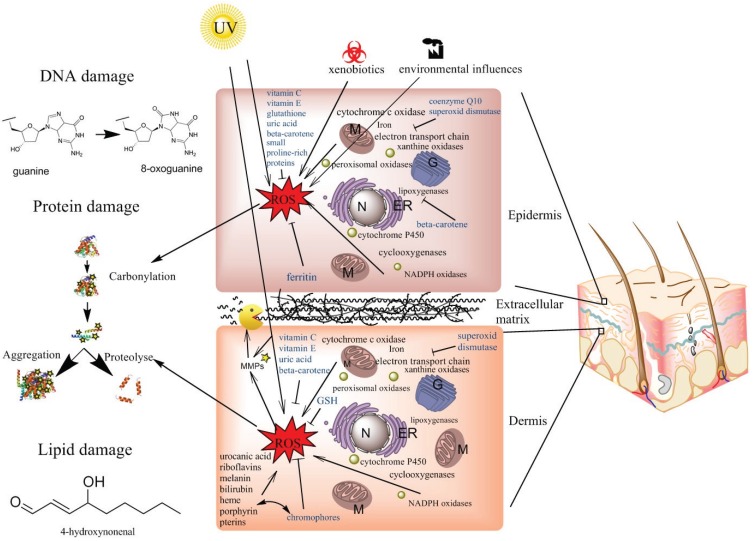
Schematic of the interplay between different ROS sources and the anti-oxidative systems in the skin. All ROS sources discussed in this manuscript are exemplified in black letters: cytochrome c oxidase, ETC (electron transport chain), iron ions, xanthine oxidase, peroxisomal oxidases, lipoxygenases, cytochrome P450, cyclooxygenases, NADPH oxidases, UV-radiation, xenobiotics and several chromophores that lead to ROS. Examples of anti-oxidative systems are given in blue letters: vitamin C and E, GSH (glutathione), GSH peroxidases, uric acid, beta-carotene, the SPRR2 (small proline rich repeat) family, SOD (superoxide dismutase), CoQ10 (coenzyme Q) and ferritin. Generally it has to be stated that only the SPRR2 proteins are specific for the epidermis, but the concentration of most anti-oxidants is much higher in the epidermis than in the dermis. Possible outcomes of oxidative damage to the cells (damage to DNA, proteins and lipids) are shown on the left-hand side of the figure. (M) stands for mitochondria, (ER) for endoplasmic reticulum, (N) for nucleus and (G) for Golgi apparatus.

The sources of ROS, enzymatic as well as non-enzymatic, in the cell are manifold. Enzymes that are ROS producing, on purpose or as a byproduct, include the mitochondrial electron transport chain, NADPH oxidases, xanthine oxidoreductase (XOR), several peroxisomal oxidases, enzymes of the cytochrome P450 family, cyclooxygenases, and lipoxygenases. All possible sources of ROS, the whole anti-oxidative system of the skin as well as all cellular damages are summarized in Figure 1.

### 4.1. Mitochondrial ROS Production

The electron transport chain resides in the inner mitochondrial membrane. The electrons are fed into complex I via NADH and into complex II via FADH_2_, then transferred to complex III and finally to complex IV. In complex IV (the cytochrome c oxidase) the electrons are finally deposited at molecular oxygen resulting in the production of H_2_O. However, before the electrons reach complex IV they can leak prematurely to O_2_ at complex I and III, leading to the formation of superoxide instead of water [2,77,78]. It is estimated that no less than 1%–2% of all oxygen consumed leads to the formation of superoxide [79]. The contribution of mitochondria to the production of ROS in the skin is only substantial in the stem cells, but further on is small compared to other organs, because during the cornification process the keratinocytes degrade all their organelles including the nucleus, mitochondria, peroxisomes, and the endoplasmic reticulum [80,81]. The importance of mitochondria in the aging process independently of the production of ROS is summarized elsewhere [82,83,84].

### 4.2. Peroxisomal ROS Production

It is still commonly assumed that mitochondria are the main contributors to ROS production in the cell, but increasing knowledge in the last decade led to the conclusion that the endoplasmic reticulum as well as the peroxisomes produce as much or even more ROS than mitochondria [85]. Peroxisomes, especially, are filled with a wide variety of enzymes, mainly flavoenzymes/oxidoreductases, that are supposed to produce hydrogen peroxide as a byproduct. All these enzymes are either involved in the β-oxidation of fatty acids, D-amino acid catabolism and anabolism, glyoxylate/dicarboxylate metabolism or the production of the autophagy stimulating and life prolonging substance spermidine. These enzymes are namely Acyl-CoA oxidase 1, 2, and 3, D-amino acid oxidase, D-aspartate oxidase, L-pipecolic acid oxidase, L-α-hydroxyacid oxidase 1 and 2, and the polyamine oxidase [85]. Furthermore, peroxisomes not only produce ROS hydrogen peroxide, but similar to mitochondria have the capacity to form superoxide (O_2_^−^). The O_2_^−^ mainly originates from the enzyme xanthine oxidase and seems to be important during ischemia reperfusion injury [86,87]. Xanthine oxidase is found in the cytosol as well as in the peroxisomes and is the terminal enzyme and therefore key player in purine degradation. The two reactions that are catalyzed by this enzyme are the hydroxylation of hypoxanthine to xanthine and the hydroxylation of xanthine to urate [88]. Besides reactive oxygen radicals, a cell can also produce reactive nitrogen species that are not discussed in detail in the course of this review. The hemeprotein nitric-oxide synthetase catalyses the oxidation of L-arginine leading to nitric oxide (NO). In the absence of this amino acid no nitric oxide but superoxide is formed [89].

### 4.3. ROS Production in the Endoplasmic Reticulum

Oxygen radicals are not only produced, in mitochondria and peroxisomes, but also in the endoplasmic reticulum. The main contributors to ROS production in this organelle are members of the cytochrome P450 family and the combination of the protein disulfide isomerase PDI and the endoplasmic reticulum oxidoreductin-1 (ERO1). The protein PDI induces the formation of disulfide bonds in receptor proteins during the folding process. The isomerase gets reduced in this process and is regenerated by the oxidoreductin ERO1. The reduced protein ERO1 finally transfers the electron via the cofactor FAD to molecular oxygen. Incomplete transfer can lead to the production of superoxide [90,91].

The cytochrome P450 family, mainly found in the ER, is responsible for the detoxification of xenobiotics or lipophilic compounds, mainly by increasing the water solubility of these substances. For this process electrons are transferred from NADPH to cytochrome P450 via the cytochrome P450 reductase, finally leading to the hydroxylation of xenobiotics. A leaky transfer of electrons can result in the formation of oxygen radicals, especially superoxide [92,93]. The main P450 enzymes that are expressed in the skin are cytochromes 1A1, 1B1, 2B6, 2D6, 2E1, 3A4, and 3A5 [94,95].

### 4.4. ROS Production in Membranes and in the Cytosol

Even membranes harness the power to produce reactive oxygen species. This is due to the activity of NADPH oxidases. Electrons are passed on from NADPH over FAD and two b-type hemes to the final acceptor O_2_, resulting in the formation of superoxide. In contrast to all other ROS sources discussed so far the superoxide produced this way in membranes is not the byproduct of catalytic processes, but superoxide is actively produced as a signaling molecule or as a “weapon” against invading microorganisms [96]. These enzymes can be found in different membranes such as the plasma membrane, the ER or mitochondria [96,97,98,99].

Finally, the cytosol has the capacity to produce ROS as a byproduct of the arachidonic acid metabolism. The enzymes cyclooxygenase and lipoxygenase both use arachidonic acid as a substrate to synthesize prostaglandin H2 and the leukotrienes, respectively. Both enzymes have the capacity to produce superoxide in the presence of NADH or NADPH [100]. The levels of arachidonic acid are relatively low in the skin, but increase in inflammatory skin diseases such as psoriasis, atopic dermatitis, and eventually aging [101,102].

The reaction of oxygen with iron ions additionally contributes to the production of ROS in the cytosol and all organelles of cells. The toxic effect of iron ions relies on the reaction of superoxide with ferric iron, resulting in the formation of ferrous iron. This process is called the Haber-Weiss reaction. In the following Fenton reaction the ferrous iron reacts with hydrogen peroxide, which on the one hand regenerates ferric iron and on the other hand produces the very reactive hydroxyl radical (OH^•^), and hydroxide (OH^−^). Both are more harmful for the cell than superoxide [103,104].

The skin is at the interface between the body and the environment and is therefore in constant contact with pollutants, xenobiotics, and UV irradiation. These exogenous factors represent the main contributor to the formation of ROS in human skin, therefore being very specific for this organ. All these factors are summarized under the term exogenous ROS. Additionally, alcohol intake, false nutrition, and physiological and mechanical stress are believed to contribute to this kind of exogenous mediated ROS production [105,106]. In addition the skin is also one of the very few organs that are in direct contact with atmospheric oxygen.

### 4.5. Photoaging or UV-Induced ROS

UV-irradiation especially leads to the genesis of ROS that are in turn main contributors to the aging of skin. To stress the importance of UV irradiation and the resulting ROS formation on the aging process of the skin the term “photoaging” has been coined. ROS production is mainly driven by UVA, in the range of 320–400 nm. The UVB light does not have the capacity to penetrate to the deeper sections of the epidermis. The UVA light induces different changes in the dermis and these seem to be mainly responsible for the process and progression of photoaging [107]. In fact, these dermal alterations are better studied than the changes in the epidermis. UVA light penetrating the skin, is on its way absorbed by cellular chromophores. These cellular chromophores involve components like urocanic acid, riboflavins, melanin, bilirubin, heme, porphyrin, and pterins, but not DNA [106,108]. These photosensitizers absorb photons/energy leading to an excited state of the chromophores called the singlet excited state. Following this initial reaction two reactions can take place: a falling back to the ground state with the emission of either heat or fluorescence or second an intersystem crossing leading to a triplet excited state. This triplet excited state is only an intermediate state that can react with both DNA and molecular oxygen resulting in either modification of DNA or production of ROS such as superoxide, hydroxyl radical, singlet oxygen, or hydrogen peroxide [108,109], which in turn leads to cellular damage discussed later in this review. The process of photoaging also effects DNA, especially the mtDNA. It was demonstrated that UV-irradiation leads to a“common”, 4977 bp long deletion of mtDNA [110] that contributes to an increased ROS production of mitochondria. This increased ROS production leads to increased levels of mtDNA damage and has in this way the potential to start a vicious cycle.

Besides UVA, UVB also contributes to photoaging. However, due to its limited penetration ability UVB acts only on epidermal cells but not on the dermis [107] and leads to damage in keratinocytes and melanocytes. Nevertheless, UVA mediated epidermal damage affects the subjacent dermis. Moreover, the near-infrared light also has an effect on the epidermis as well as the dermis. It was shown that infrared light is absorbed by the mitochondrial ETC, especially at complex IV, leading to an increased leakage of ROS into the mitochondrial matrix. Krutman and Schroeder [107] introduced the term of the “defective powerhouse model”. In this model UV as well as infrared light leads to an impaired energy production by dermal mitochondria that alters the morphology and function of the skin via retrograde signaling. Although the minor penetration ability of UVB is an advantage for skin aging, it has the big disadvantage that it does not act via photosensitizers but can directly damage the cell and DNA [106].

Amongst a variety of xenobiotics and pollutants that have the capacity to induce ROS production in the skin, polycyclic aromatic hydrocarbons are of special interest. These planar aromatic compounds are found in coal, oil, and tar and are especially dangerous after burning [111]. After absorbing the energy from light these substances reach a photo-activated state and react in subsequent processes with molecular oxygen under production of ROS [112].

## 5. Anti-Oxidative Capacities of the Skin

### 5.1. Anti-Oxidative Properties of the Cornified Envelope

To cope with these many sources of ROS the skin has developed sophisticated and in part very skin-specific anti-oxidative mechanisms. Most of the anti-oxidants show in fact a higher concentration in the epidermis than in the dermis [113]. This correlates well with the fact that the ROS load is higher in the epidermis than in the dermis. The epidermis is built up in a very gradual way and displays an increasing calcium concentration from the *stratum basale* to the *stratum granulosum* where a peak is reached. Also the cornified envelope gradually increases in its density. The formation of the cornified envelope starts in the *stratum spinosum* and is fully assembled in the *stratum corneum*. An epidermal concentration gradient is also found in the case of anti-oxidants, especially the low-molecular-weight ones. Vitamin C, vitamin E, glutathione, ubiquinol, and uric acid are detectable in the *startum corneum*, but their concentration increases steeply towards deeper cell-layers of the *stratum corneum* [105,114]. These comparably low concentrations of non-enzymatic and lipophilic anti-oxidants in the outer layers of the *stratum corneum* are possible, because the cornified envelope itself has anti-oxidative capabilities. These anti-oxidative capabilities of the cornified envelope rely on the SPRR proteins. Members of this protein family are not only rich in prolins but have an over-proportional enrichment incysteines. Therefore these proteins can quench ROS by forming intramolecular disulfide bonds. Interestingly these anti-oxidative properties were mainly found for the SPRR2 subfamily. This fact can be explained by different accessibilities of the cysteine residues of these cornified envelope proteins [113]. According to Harman’s idea [2] ROS levels increase in the aging process. Indeed, we have found that the CE is dramatically altered in the aging process. Based on our own work [72] we argued that the loss of loricrin is compensated by increased levels of SPRRs. The biggest changes were found for the SPRR2 subfamily. In the light of the anti-oxidative capacities of the cornified envelope this increase in SPRRs during the aging process represents a valid tool to cope with the increasing ROS levels during aging. Below the *stratum corneum* another, upside-down gradient of anti-oxidative substances and enzymes is found. In this gradient the highest concentrations of enzymes and anti-oxidants are found in the *stratum granulosum* constantly declining towards the *stratum basale* [115]. In this way the suprabasal cells have lower ROS levels and are protected against UVB-induced apoptosis [116]. The importance of the CE as an anti-oxidant/UV barrier is also stressed by the fact that UV can completely deplete the *stratum corneum* of anti-oxidants/vitamins [117]. Therefore only the remaining CE proteins (mainly SPRR2 subfamily) can exert their anti-oxidative properties and protect the epidermal cells.

### 5.2. The Non-Enzymatic Anti-Oxidants Vitamin C, Vitamin E, Beta-Carotene and CoQ10

The strong anti-oxidant L-ascorbate/vitamin C cannot be synthesized by primates and therefore has to be taken up with food [118]. The water soluble vitamin C itself is an electron donor and is used as a cofactor for enzymatic reactions such as the crosslinking of collagen. Vitamin C is very prominent and the most abundant of all anti-oxidants [106]. In addition this anti-oxidant can react with a potential dangerous free radical and can donate its electron. In this way vitamin C itself is oxidized and forms so called “semidehydroascorbic acid”. The big advantage of the resulting radical is that it is stable and comparably unreactive. This radical can either be reduced back or can react further to dehydroascorbic acid [119]. It was shown that vitamin C has a strong effect on photoaged skin, most probably by quenching ROS that originate from UV-irradiation [120]. It was found that the amount of ascorbate decreases in both intrinsic skin aging as well as extrinsic aged/photoaged skin [121].

The second vitamin with anti-oxidative capacities is α-tocopherol/vitamin E. Vitamin E is more than one compound, but the most important one in humans is α-tocopherol. Similar to vitamin C α-tocopherol has a very important photoprotective and anti-photoaging role in the skin [122]. In contrast to the water soluble vitamin C, vitamin E is lipophilic and is found in animal membranes. It can be nutritionally supplied by plant oils. The anti-oxidant α-tocopherol is highly important because it can stop ongoing lipid peroxidation, by the reduction of the lipid peroxyl radical to hydroperoxide. In the course of this detoxification process this anti-oxidant loses a proton and is itself transformed into a radical. However, the α-tocopheroxyl radical is not very reactive and is further on detoxified by ascorbate, glutathione, or enzymes [123]. Recent literature also indicates that α-tocopherol exerts its photoprotective and anti-aging functions not only via its anti-oxidative role but also due to its role as activator/mediator of different signaling pathways. It has, for example, been shown that the protein kinase C pathway is affected by vitamin E [124]. In the aging process the levels of α-tocopherol are unaffected in the dermis, whereas a clear decrease of this anti-oxidant was observed in the epidermis [121]. Cutaneous application of vitamin E ameliorates photoaging, decreases lipid peroxidation and furthermore also reduces photocarcinogenesis, MMP-1 transcription levels and thymine dimer formation [106].

Beta-Carotene is produced by plants and bacteria and also has to be taken up by food. This substance is a provitamin for retinol. In addition it has been shown that this precursor of vitamin A has a huge effect on skin aging and photoaging by either scavenging radicals or inhibiting lipoxygenases that are capable of producing ROS as discussed above [125,126]. Beta-carotene, a typical skin carotene, is anti-oxidative because the peroxyl radical is directly added to its backbone forming an epoxide that is decomposed afterwards [127].

The next anti-oxidant discussed here is somehow ambiguous. Uric acid on the one hand is the final product of the degradation of purines and is created by an enzyme that itself is capable of producing ROS as discussed above. On the other hand it is an anti-oxidant. Similar to ascorbate, uric acid is a reductant for ROS and can scavenge radicals such as hydroxyl radicals, singlet oxygen, and oxo-heme oxidants. By absorbing one electron, uric acid itself is transformed into a radical, although not very reactive [128]. It was also shown that uric acid is the main anti-oxidant in serum [129]. Therefore the contribution to the anti-oxidative capacity of the skin is comparably low as the skin has a low blood supply. Moreover, it was demonstrated that the extracellular urate is a potent anti-oxidant but acts as a pro-oxidant within the cell [130].

The last enzyme-free anti-oxidant discussed in this review is CoQ10. CoQ10 is known because of its contribution to the mitochondrial ETC. Ubiquinone is reduced to ubisemiquinone and ubiquinol at complex I and II and oxidized back to ubiquinone at complex III [131]. Besides this important contribution to the ETC, ubiqinone has also been described as an anti-oxidant. The lipid soluble CoQ10H2 is a chain breaker in lipid peroxidation and protects lipids from lipid peroxidation [132]. In comparison to vitamins C and E, ubiquinone seems to be ineffective in photoprotection [132].

### 5.3. The Importance of Superoxide Dismutases, Catalases, Glutathione Peroxidases, Ferritin, and Peroxiredoxins in Quenching ROS

Among the most prominent enzymes that can handle reactive oxygen species are the superoxide dismutases. These enzymes “dismutate” superoxide to hydrogen peroxide [133]. In mammals three isoforms can be distinguished that differ in their localization. The enzyme SOD1 is found in the cytosol and nucleus and has Cu/Zn as cofactor, SOD2 is found in mitochondria to dismutate superoxide originating from the mitochondrial ETC and binds Mn^2+^, and SOD3 is found in the extracellular space harboring the metal ions Cu/Zn in its active center [134]. In the first half-reaction the electron from the superoxide radical is transferred to the metal ion in the active center thereby reducing it. The superoxide itself is oxidized to O_2_. In the second half-reaction the reduced metal in the superoxide enzymes is reoxidized by transferring the electron to superoxide resulting in the formation of hydrogen peroxide [135]. All three human superoxide dismutases have a huge impact on aging skin. Generally a deletion of superoxide dismutase is lethal as demonstrated in mice, but with SOD mimetics life can be prolonged for several weeks. *Sod1*^−/−^ mice show a clear skin atrophy that is also observed in aged individuals [136,137]. In case of SOD2 deletions the phenotypes are even more dramatic. UV irradiation leads to the above discussed mtDNA deletions and results in a burst of radicals from the defective mitochondrial ETC. Not surprisingly, UV irradiation results in a dose dependent increase in SOD2 mRNA levels in wildtype mice [138]. Although SOD2 overexpression had no obvious life prolonging effects [139], distinct skin aging phenoytpes were observed in *Sod2*^−/−^ mice. These phenotypes comprise a thinning of the epidermis, a clear atrophy of the dermal connective tissue, a reduced amount of procollagen I, and an atrophy of the subcutaneous fat tissue [140,141,142]. The SOD3 enzyme is expressed in the dermis as well as in the epidermis. By harboring a heparin-binding domain this enzyme is in close contact with the extracellular matrix and cell surfaces. In contrast to SOD1 and SOD2 very high doses of UV are needed to induce the expression of SOD3. Therefore the role of SOD3 in the skin is unclear although it has been shown that SOD3 is involved in skin inflammation and its expression is reduced in psoriasis [134,143].

A very prominent enzyme that detoxifies hydrogen peroxide is the peroxisomal localized catalase [144]. This enzyme consists of four identical polypeptide chains, each harboring a heme group [145]. In a first step hydrogen peroxide reacts with the heme group leading to an oxoferryl porphyrin cation radical and a water molecule. The so called compound I is very active and reacts immediately with a second hydrogen peroxide molecule producing water and molecular oxygen and regenerating the original prosthetic heme group [146]. The catalase enzyme is very prominently expressed in the skin, especially in the *stratum corneum*. The amount of catalase exceeds the amount of superoxide dismutases. Inside the *stratum corneum* a gradient of activity, with a decreasing activity towards the surface of the skin, was detected [147,148]. In the aging process the activity of this enzyme is altered with a widening gap between the dermis and epidermis. Thus, catalase activity decreases in the dermis and increases in the epidermis of aged and photoaged skin. Because the ROS load of the cells, especially in the epidermis, increases with aging, increasing catalase activity is reasonable, whereas the reduction of catalase in the dermis remains mysterious [148,149]. A remarkable experiment showed that by targeting the peroxisomal catalase to mitochondria a statistical significant increase in medium and maximum lifespan was found in mice [150].

A main contributor to the anti-oxidative potential of the cell is the tripeptide glutathione GSH, harboring a special gamma peptide linkage. This peptide is synthesized in a two-step process. The first step is performed by the gamma glutamylcysteine synthetase, the second step by the glutathione synthetase. The GSH acts as an anti-oxidant because of its thiol group. In the course of the process GSH is oxidized by reactive oxygen radicals and forms a dimer with another activated GSH via formation of a disulfidic bond (GSSG). GSH can be recovered in a reducing step by the glutathione reductase consuming NADPH [151]. GSH not only detoxifies ROS, but can also regenerate oxidized α-tocopherol and retinol [106]. In aged mice it was shown that both, the absolute amount of GSSG as well as the GSSG:GSH ratio strongly increases in the dermis in comparison to young skin [152]. In photoaged skin the concentrations of glutathione are reduced, but this effect could be compensated by an increased activity of the glutathione reductase [121]. It is estimated that in aged skin the concentration of anti-oxidants is strongly decreased, in line with this the levels of α-tocopherol, ascorbate and GSH have been shown to be reduced by 70% [121]. The function and (inter)action of all anti-oxidants is deeply interwoven to keep the redox state in the skin tissue in balance. For example, vitamin C can reduce oxidized α-tocopherol and is itself oxidized; glutathione in turn can rescue vitamin E and the resulting GSSG is converted into GSH again by the glutathione reductase enzyme [106].

Beside its role as an anti-oxidant, GSH is also a cofactor for enzymatic reactions. The glutathione peroxidase is an enzyme that fulfills two tasks: reduce hydrogen peroxide to water and stop lipid peroxidation. In humans, eight glutathione peroxidases are known, five of them containing selenium as a co-factor. In a first step, the peroxide, either lipid or hydrogen, oxidizes the enzyme bound Se, thereby forming SeOH. In a next step the enzyme reacts with a thiol group in GSH resulting in the formation of a selenylsulfide bond between the enzyme and the glutathione. A reaction with a second GSH regenerates the enzyme and GSSG is formed [153,154]. Alterations in the enzyme activities in aging skin and photoaged skin have not yet been characterized, however a targeted disruption of the glutathione peroxidase 4 in mice displayed severe skin phenotypes, like hyperplasia of the epidermis, dermal inflammation, increased rates of lipid peroxidation, and higher levels of the cyclooxygenase-2 [155].

As already mentioned, free iron ions are a constant threat for the cell because the Fenton reaction is capable of starting a vicious cycle of ROS production in the cell, ultimately leading to its death. Therefore the cell has to conceal the iron ions very carefully. This iron storage is achieved by the protein ferritin. The protein consists of 24 subunits forming a sphere that surrounds the iron. The iron is only stored in its Fe(III) form as ferrihydrite and upon its release it has to be reduced to the Fe(II) form. The 24 subunits can be divided into two subtypes: the heavy (H)-type and the light (L)-type. The L-type is involved in the core-formation, the H-type in the Fe(II) oxidation [156]. Ferritin is primarily stored in the cytosol, although mitochondrial and nuclear forms are also known. The iron release is also dependent on lysosomal ferritin degradation [157]. Ferritin seems to be an important tool in the regulation of the redox homeostasis especially after UV irradiation. The highest concentrations of ferritin in the skin are found in the *stratum basale* [158]. The levels of ferritin in the epidermis are around three to seven fold higher than in the dermis. After UV irradiation, especially UVA, the levels of ferritin in the dermis as well as epidermis, strongly increase, indicating a potential anti-oxidative mechanism to stop ROS production in cells after disturbing the redox homeostasis [158,159]. However, the combination of iron storage, ferritin, and UV irradiation also has detrimental potential. It was demonstrated that UVA irradiation of primary dermal fibroblasts induces an immediate degradation of ferritin in the lysosomes, followed by a release of iron ions into the cytosol accompanied by a burst of ROS [160]. The acceleration of skin aging in females after the menopause was also attributed to iron and ferritin. In females there are two ways to get rid of excessive iron: menstruation and desquamation. After the menopause the excessive iron ions are stored in the skin via ferritin and this could contribute to an increase in ROS levels that accelerates the aging process in the skin [161].

The last class of enzymes that have anti-oxidative capacities and are discussed in this review are the peroxiredoxins. In mammals six isoforms were identified, whereas 2-Cys enzymes (PRDX1-5) and 1-Cys enzymes (PRDX6) can be distinguished [162]. In the following, only the 2-Cys enzymes will be discussed. A peroxide substrate reacts with a conserved cysteine in the active center of these enzymes leading to the formation of a sulfenic acid residue. This is followed by a reaction of the sulfenic acid with a second cysteine (therefore the term 2-Cys enzyme), thereby forming an intra-molecular disulfide bond [151]. The enzyme is regenerated by a flavoprotein disulfide reductase such as the thioredoxin reductase [162]. More details of the function of this enzyme can be found in the Chapter “Oxidative stress in fungi” in the same issue. High levels of PRXD1-3 were found especially in the epidermis but also in the dermis of rats. Similar to the calcium distribution, PRDX1 and PRDX2 were found with increasing concentrations towards the *stratum granulosum*. PRDX3 showed the opposite distribution. The highest concentration was found in the *stratum basale*, the lowest concentration in the *stratum granulosum* [163]. The peroxiredoxins seem to be very important in the detoxification of ROS originating from UV irradiation. UVB-irradiation induced the expression of PRXD2, UVA the expression of PRDX1 [163,164]. Overexpression of PRDX6 leads to significantly reduced levels of oxidized lipids in mice and results in a reduced rate of UVB and UVA induced apoptosis, whereas loss of PRDX6 leads to an increased skin tumor rate [165,166]. There is also growing evidence that an increase of activity of peroxiredoxins has great potential in increasing lifespan [167].

### 5.4. The Anti-Oxidant Treatment Paradox

An increase in ROS levels over time is a common feature of all human tissues and especially of the skin. Therefore many attempts were made to quench these ROS by topical treatment of the skin or supplementation with anti-oxidants, in the hope to improve or even rejuvenate aged skin. But the results are very controversial and are heavily disputed in the literature. Just recently it was demonstrated that a mixture of alpha hydroxy acids, vitamins B_3_, C, and E applied on facial skin dramatically improves the quality of the epidermis and dermis including the smoothening of wrinkles and the refinement of skin texture without side effects [168]. A similar effect was found on treating aged and photodamaged skin with a special combination of several anti-oxidants consisting of resveratrol, baicalin, and vitamin E. These antioxidants were partially sufficient to rejuvenate aged skin [169]. Resveratrol was shown to stimulate the Nrf2 pathway in skin leading to an increase in the GSH content and improvement of skin quality [170]. Also CoQ10 conjugated with nanoparticles (to improve the skin permeability) seems to have a beneficial effect on skin quality [171,172]. Some additional substances that seem to have a positive effect on aged skin, especially the epidermis, are summarized in Lorencini *et al.* [173]. Though many studies promote the use of antioxidants for preventing skin aging, others warn of potential side effects. Treatment of various model organisms with vitamin C gave a broad variety of results ranging from a prolonged lifespan to “no effect” [174]. Surprisingly, no beneficial, statistically significant effect on lifespan elongation was found for food supplementation with vitamin E [175,176]. In the small rodent *Microtus agrestis* supplementation with vitamin C and E led to a remarked reduction of lipid peroxidation, as expected, but significantly reduced the lifespan of this organism [176,177]. Also CoQ10 can lead to both, an increased (mice, nematodes) or decreased (*S. cerevisiae*) lifespan [176,178,179,180]. Recent literature also warns of the excessive use of vitamins. Oral administration of beta-carotene, vitamins E and A in humans seems to lead to a higher mortality rate [181] or increased risk of diseases [182]. Quite surprisingly it was shown that increased ROS levels and increased oxidative damage can even lead to an increase in lifespan [183,184]. This controversy can be explained by the fact that ROS are not exclusively detrimental for cells, but can even be beneficial. There is growing evidence that ROS, especially hydrogen peroxide, have an important role in cells as a second messenger [96,185]. Therefore it is not desirable to quench away all the ROS, because this influences the ROS homeostasis of the cell with such detrimental effects as promoting tumor formation [186]. A beneficial effect on skin aging by a treatment with antioxidants can onlybe achieved if the original ROS level of healthy cells is preserved.

## 6. Protein Oxidation, Lipofuscin and AGEs

ROS originating from all sources discussed in the previous chapters basically affect all compartments of the cell. While also attacking DNA and lipids, the modification and potential subsequent aggregation of proteins poses a major problem for cells. As described above, especially UV exposure significantly increases ROS levels in skin cells [187,188]. It has been shown that protein oxidation and detectable unfolding occurs after only 30 min of UV exposure in human skin [189]. Continued UV irradiation accompanied by ROS production over longer periods of time is especially problematic and results in more pronounced modifications like the aggregation of unfolded proteins [190].

ROS can modify proteins directly or indirectly. Indirect attacks come from secondary by-products. An example of this is the protein backbone fragmentation that occurs after previously oxidized glucose binds to amino groups. This may be interesting in the context of *diabetes mellitus* since there is evidence that this disease is accompanied by increased oxidative stress. Backbone fragmentation could partially account for the tissue damage associated with it [191,192].

Direct ROS protein modificationsare reported at the backbone, at amino-acid side chains or by the formation of carbonyls. Hydroxyl-radicals (^•^OH) may initiate backbone damage by abstracting hydrogen atoms from the α-carbon of polypeptide chains. This initiates a chain of reactions that ultimately lead to the formation of alkoxyl derivatives resulting in spontaneous cleavage of the derivate itself [193]. Several amino acid residues are more susceptible to oxidative modifications than others. Examples of that are histidine, leucine, methionine, and cysteine as well as phenylalanine, tyrosine, and tryptophan. Only modifications of the sulfur-containing amino acids methionine and cysteine are reversible, for example by the enzymes glutaredoxin 1, thioredoxin, and methionine sulfoxide reductases [194,195,196].

### 6.1. Protein Carbonyls

As mentioned above modifications of other amino acids form more stable products like carbonyl groups [197]. For example, oxidation of protein side chains containing proline, arginine, lysine and threonine results in the formation of carbonyl (CO) groups (aldehydes and ketones). These modifications are therefore irreversible and serve as early markers of oxidative stress [198]. The stability of these protein modifications has led to the general opinion that they are more than markers though. The introduction of carbonyl groups induces conformational changes of the polypeptide chain and leads to partial or total inactivation. This would make protein carbonyls main contributors to the detrimental effects arising from oxidative stress. At the very least the increased formation of protein carbonyls may overburden the cell’s repair/degradation system resulting in an accumulation of more modified and inactivated proteins [199]. Suntanned epidermis shows a considerably higher amount of carbonylated proteins than epidermis that is rarely exposed to sun and therefore UV-irradiation [200]. Besides UV exposure acrolein, a component of cigarette smoke, was shown to play a significant role in protein carbonylation, as shown in keratinocyte cell culture experiments [201]. Investigating the level of carbonyl groups in keratins in human skin, Thiele *et al.* were able to show that they were significantly higher in the *stratum corneum* as compared to deeper layers of the epidermis [202]. Considering that the *stratum corneum* is the first barrier between the body and the environment, these findings are not surprising. In this context it is also worth mentioning that proteins can be more or less susceptible to carbonylation. The reasons for this are not clear at the moment, but possibly include immediate proximity to ROS generating sites or the presence of transition metals in these proteins [203]. Additionally it has been proposed that carbonylation may play a role in ROS signaling [204].

### 6.2. The Process of Protein Oxidation

As described above, sustained UV-exposure leads to a higher extent of protein oxidation. This increased protein oxidation has also been reported in aged tissue and senescent cells. One reason for the high amount of oxidative damage in old cells is that they have been exposed to a multitude of stressors and have accumulated oxidative damages to DNA, proteins and lipids. This accumulation of oxidative damaged DNA, protein and lipids in turn results in an increased ROS production, leading to a negative feedback loop. Moreover, the ability of aged cells to efficiently clear ROS is impaired [205,206], resulting in an imbalance between the production and the clearance of ROS where more and more proteins begin to aggregate. Postmitotic cells are not able to dilute their protein aggregates by cell division [207]. Jung *et al.* were able to show that the amount of oxidized proteins is much higher in *in vitro* aged, senescent fibroblast cells compared to young cells. Experiments with long term exposure to H_2_O_2_ led to increased levels of protein oxidation in young fibroblasts. Interestingly this exposure was not sufficient to increase the already much higher protein oxidation levels of senescent fibroblasts [208]. In line with these findings, a significant increase in protein oxidation was reported in human patients’ papillary dermis and in the *stratum corneum* after daily UVB exposure for 10 subsequent days with a solar simulator, mimicking UV exposure of a typical summer vacation [189]. In general it seems that most epidermal layers are less affected than dermal layers, most probably due to the high amount of anti-oxidants in the epidermis [209] and the anti-oxidative functions provided by SPRRs [113]. This was confirmed *in vitro* with both, fibroblast and keratinocyte cell cultures. Fibroblasts showed a significantly higher amount of protein oxidation compared to keratinocytes when exposed to UVA/UVB irradiation [189].

The process of protein oxidation can be divided into various stages according to the severity of modifications. At first, only slight oxidations take place leading to a marginally reduced enzyme activity of affected proteins or changes in thermostability. In this stage no extensive unfolding takes place [210] and the cell is still able to counteract by repairing the damages. An important protein in this context is thioredoxin, the cells major protein disulfide reductase. Among other things it has an important role in the protection against oxidative stress since it prevents intra- and intermolecular formation of disulfide bonds which would otherwise lead to the inactivation and/or aggregation of these proteins [194]. Thioredoxin is also able to function as an electron donor for thioredoxin peroxidases or peroxiredoxins. Next to glutathione peroxidases and catalase, these enzymes are able to catalyze the reduction of H_2_O_2_, a major contributor to cellular oxidative stress [211].

The next stage is marked by increased protein oxidation. Due to high amounts of ROS or other sources of “protein modifiers” and the inability of the cell to clear these damages, various protein modifications are able to accumulate. The chemically modified proteins completely lose their activity and unfold extensively. Due to the unfolding proteins begin to cross-link and form small aggregates. At this point the proteins either become targets for degradation by the proteasome [197,212] or can be refolded and rescued by heat shock proteins. It is unclear how the cell decides to either refold or degrade these proteins since both possibilities have their advantages [213,214]. The inner proteolytic chamber of the proteasome is mainly accessible to unfolded proteins, a typical fate of oxidized proteins. As long as the protein is not extensively oxidized it is an ideal substrate for the 20S proteasome [213]. In contrast to this, the main substrates for the 26S proteasome are not unfolded but ubiquitinated proteins. It is a major key player in the unfolded protein response (UPR) initiated by the ER [215,216,217]. Disassembly of the 26S proteasome during age or prolonged stress leads to an increase in 20S proteasome abundance. While this may lead to an increase in the degradation of un-ubiquitinated oxidized proteins or smaller protein aggregates, ubiquitinated proteins begin to accumulate triggering different stress responses including lysosomal uptake [218,219]. On the other hand inactivation of the 20S proteasome by UVA in dermal fibroblasts leads to an activation of activator protein-1 (Ap-1), controlling MMP-1 (matrixmetalloprotease-1) expression [220]. As mentioned above MMP-1 is responsible for increased extracellular protein degradation [221,222].

The last stage of protein modification is marked by extensive oxidation of proteins, their complete unfolding and covalent crosslinking of several proteins [223,224,225,226]. Oxidized, unfolded proteins begin to form extensive, insoluble aggregates due to interactions between the exposed hydrophobic residues [213] and later on form covalent bonds. Not only are these aggregates poor substrates for the proteasome due to their sheer size, they are also able to cause proteasomal inhibition [227,228]. A reason for that may be the extensive crosslinking of the various proteins in the aggregate. The proteasome cannot degrade these aggregates due to steric/mechanic inhibition and remains bound to the structure, unavailable for other substrates [229]. In addition to that, the proteasome loses efficiency in age resulting in even more accumulation of modified proteins [230,231]. It was demonstrated that the proteasomal activity decreases significantly beginning in middle aged (60 ± 8 years) skin samples, especially in the dermis. Surprisingly, no further decrease in proteasome activity was observed in older skin donors (90 years) [232].

### 6.3. Lipofuscin

Continued elevated ROS levels eventually lead to the formation of large protein-lipid aggregates known as lipofuscin. These structures can be detected in nearly all types of cells including fibroblasts and keratinocytes [197,233,234]. Lipofuscin (meaning “dark fat”) appears as yellow-brownish material in the light microscope and shows autofluorescence over a broad spectrum [207]. It is also known as “ceroid” or “age pigment” [235,236]. The term “age pigment” is especially interesting in the context of the skin since the formation of lipofuscin gradually increases with the age of the individual. In old individuals lipofuscin has accumulated excessively and becomes visible with the naked eye, hence the term “age pigment” [236,237]. Lipofuscin eventually also incorporates lipids and forms protein-lipid clusters, consisting of 30%–70% proteins and 20%–50% lipids [238]. In very old individuals lipofuscin clusters begin to incorporate sugar residues [239]. Lipofuscin clusters have the capability to bind various metals like copper, zinc, manganese, calcium and iron [240] as well as metal-containing proteins like ferritin [241]. These metals can amount to 2% of the final volume of the lipofuscin cluster [212] and the irreversible binding of these metals makes these clusters another redox-active site for the generation of radicals such as the hydroxyl radical (^•^OH) [242]. This massive radical formation catalyzing structure in the cell and the inhibition of the proteasome start a vicious cycle leading to even higher amounts of oxidized proteins and eventually protein aggregates [208]. In motor neurons lipofuscin can occupy up to 75% of the cell’s volume decreasing its functionality and later on leading to apoptosis [243].

As mentioned before, mitochondria, the endoplasmatic reticulum and peroxisomes are main contributors to intracellular ROS levels and mainly target the cytosol. The same can be said for externally applied ROS contributors like xenobiotics and UV-irradiation that play a big role in skin. The oxidation of cytosolic proteins could be a kind of “buffer” before nuclear proteins are oxidized [244,245]. It has to be mentioned though that chronic or repeated UV and ROS exposure still leads to the accumulation of nuclear protein oxidation that is able to “block” the nuclear proteasome. An oxidation of nuclear proteins has been shown in senescent fibroblasts and young fibroblasts chronically stressed with appropriate chemicals like H_2_O_2_ or paraquat [208]. It looks like the carbonylated proteins in the nucleus are either excluded quite efficiently or are not sufficient for the formation of lipofuscin. It has been shown in fibroblasts that most of the lipofuscin can be found in the cytosol with a major amount of it inside the lysosomal lumen [208]. Lysosomes are primarily responsible for the removal and degradation of lipofuscin and contain a high amount of hydrolytic enzymes such as proteases, nucleases, lipases and phosphatases [246]. Surprisingly it has been proposed that besides inhibiting the proteasome, lipofuscin may also be able to inhibit lysosomal proteases [227]. It is also not quite clear if the lysosome itself may play a role in the formation of larger lipofuscin clusters. Extensive cross-linking between proteins in aggregates could also take place inside lysosomes, making them mandatory for the formation of advanced protein-lipid clusters. However Höhn *et al.* were able to show that lipofuscin also forms in dermal fibroblasts when blocking the lysosomal uptake. The formation of lipofuscin was accompanied by elevated levels of ROS [247].

One of the proposed uptake mechanisms into the lysosome is macroautophagy [248], leading to the establishment of the term “aggrephagy” [249]. During macroautophagy, portions of the cytosol as well as organelles are swallowed by a double-membrane vesicle called autophagosome. The autophagosome begins to form at an isolated membrane called phagophore although it is still not clear what the origin of this membrane is. The autophagosomal membrane then fuses with the lysosomal membrane. Its cargo is then released into the lysosomal lumen for degradation. The fusion of the two membranes also results in a bigger lysosomal compartment [250,251]. In addition to macroautophagy, microautophagy [252] as well as chaperone-mediated autophagy [253,254] are possible candidates for the removal of oxidized proteins and lipofuscin. While microautophagy involves the direct uptake of cytosolic components and organelles into lysosmes [255] chaperone-mediated autophagy specifically targets proteins containing the sequence signature “KFERQ” [256].

Autophagic uptake and transport to the lysosome is the most efficient way of removing/containing oxidized proteins after aggregates have formed. This is especially important for old, postmitotic cells which cannot dilute their protein aggregates through cell division [207] and where the proteasome has lost efficiency and is even more inhibited by the formation of lipofuscin [230,231]. Nevertheless, the progressive oxidation of proteins severely impacts the cell’s metabolism, even in younger cells. Since protein aggregates provide a redox-active surface the increased generation of ROS also elevates the oxidation levels of DNA and lipids. In addition several disorders are known or were proposed to be associated with protein aggregation. Examples are neurological disorders like Alzheimer’s and Huntington’s [257,258] as well as skin disorders like cutaneous amyloidosis and Darier disease [259,260].

### 6.4. Advanced Glycation End Products

The formation of structures known as advanced glycation end products (AGE) is another problematic process that can be significantly accelerated by oxidative stress. AGEs originate from the non-enzymatic glycation reaction between sugars and proteins, nucleic acids or lipids. The starting point of AGE formation is the Maillard reaction in which carbonyl groups of sugars react with proteins, lipids, or nucleic acids resulting in an unstable Schiff base [261]. Reorganization then leads to the formation of more stable ketoamins (Amadori product). While Schiff bases and Amadori products are reversible they have the ability to react with amino, sulfhydryl, and guanidine groups in proteins [262]. These reactions form protein adducts and protein crosslinks and give rise to AGEs [263]. In addition further oxidation by ROS or oxidative breakdown can lead to more diverse products called advanced glycation end products [264].

AGEs are a very heterogeneous group of molecules and can either be ingested through food consumption or formed inside the cell [265]. It has been confirmed that AGE deposits are accompanied by autofluorescence of the skin depending on their composition and the aging process in general [266] and that they are related to several diseases [267,268]. Interestingly the cell has specific receptors for AGEs (RAGE). Stimulation of these receptors leads to an activation of several pathways cumulating in the activation of the transcription factor nuclear factor kappa-B (NFкB). This factor increases the transcription of pro-inflammatory genes and RAGE themselves leading to a vicious cycle [269]. It was shown that RAGEs are highly expressed on mRNA as well as on protein level in fibroblasts and keratinocytes and that expression was increased in sun-exposed skin [270].

Since AGEs are able to react with a great variety of biomolecules, consequences of their formation are manifold [271]. Examples are collagen crosslinks that lead to decreased flexibility [272], the modification of intracellular proteins like cytokeratin 10 in keratinocytes [273] and functional alterations in low-density lipoprotein [274]. AGEs are also heavily connected to oxidative stress [275,276]. RAGE signaling can directly induce oxidative stress by decreasing the activity of superoxide dismutase (SOD) or indirectly by reducing cellular anti-oxidant defenses [269,277]. Due to induction of fibroblast activation, the crosslinking of collagen and the increase in metalloproteinase production (MMP 1, 2, and 9) AGEs severely affect the dermis [278]. Concerning the epidermis it was proposed that AGEs impair the migratory and proliferation abilities of keratinocytes *in vitro* [279]. In addition to these problems there is evidence that the removal of AGEs poses a big problem for the cell. As mentioned before AGE accumulation correlates well with the age of the individual [266] and seems to be resistant to proteolytic degradation [280]. There are enzymes in place to counter the genesis of AGEs [271]. One such enzyme is glyoxylase I which removes α-dicarbonyl compounds, another starting point for AGEs. Unfortunately though the decreased activity of this enzyme has been reported during aging [281]. All of these facts paint a very complex picture of the origin and impact of advanced glycation end products. The mutual interactions between AGEs and ROS, induced for example by UV irradiation, make it a challenging area that still needs a lot of further investigation.

## 7. Oxidative Stress, DNA, Cancer and Senescence

### 7.1. DNA Mutation

Besides the already discussed “common” mtDNA deletion, nuclear DNA is also affected by ROS. Of great importance for the oxidation of DNA is the hydroxyl radical originating from the Fenton reaction. The reaction of ROS with other free radicals leads to a multitude of DNA base products that are potentially mutagenic. These DNA base products are summarized in great detail in Cook *et al.* [282]. The most familiar of these ROS-induced DNA alterations is 8-oxo-2'deoxyguanosine which pairs with an adenine as well as with a cytosine resulting in a GC to TA transition [283]. It was shown that this mutation accumulates specifically during aging in skin and basically all tissues studied [284,285,286]. Besides the modification of DNA bases, ROS also induce single-strand breaks and to a lesser degree double strand breaks [287]. UV irradiation not only induces ROS production in skin cells and in this way leads to DNA damage but can directly affect DNA. Especially UVB irradiation leads to the formation of cyclobutane pyrimidine dimers (CPD) and (6–4) photoproducts (both from thymine or cytosine bases). C-C dimers as well as the less abundant but more mutagenic (6–4) photoproducts induce a GC to TA transition, [288,289,290]. The (6–4) photoproducts are removed primarily by the nucleotide excision repair (NER), whereas the most important repair mechanism for cyclobutane pyrimidine dimers is not the NER but the CPD photolyase. Another photolyase exists for the (6–4) photoproducts [291,292,293,294]. The ROS induced small DNA damages such as 8-oxo-2'deoxyguanosine, abasic sites and tymine glycol are predominantely repaired by the base excision repair (BER) and the very rare double strand breaks by non-homologous end joining [285,295]. Defects in the nucleotide excision repair lead to severe human diseases that have one thing in common: a segmental premature aging phenotype. In sum 11 diseases were identified that are based on defects in the NER. Besides Cockayne syndrome and trichothiodystrophy, the skin disease Xeroderma pigmentosum has to be named. *Xeroderma pigmentosum* does not only lead to segmental progeria but also to a dramatic increase in sun sensitivity and an increased incidence of sun-induced skin cancer [296,297]. During the aging process a clear decline in the NER as well as the BER could be observed. Sauvaigo *et al.* tested the repair capacities of primary fibroblasts obtained from three different age groups: young adults (mean age: 25 years), middle-aged adults (mean age: 46 years) and old adults (mean age: 65). The capacity of NER in repairing cyclobutane pyrimidine dimers clearly decreased for the old individuals. Surprisingly the capacity of BER to repair DNA damage seems to decrease much earlier. The repair efficiency for 8-oxo-2’deoxyguanosine, abasic sites and thymine glycol was dramatically reduced in the old as well as the middle aged group [284]. This decrease in repair capacity could explain the increased prevalence of old people for skin cancer. 

### 7.2. Cancer

Superoxide is very often considered as the ‘‘primary’’ ROS, because it can be metabolized to other even more reactive radicals such as the hydroxyl radical [298]. The hydroxyl radical can react with the guanosine in the nucleotide chain under formation of 8-oxo-2'deoxyguanosine [299]. In this context it is not surprising that ROS are important factors in tumor development and high levels of 8-oxo-2'deoxyguanosine are found in a multitude of cancers [298,300,301,302,303,304]. The three most frequent skin cancers are basal cell cancer, squamous cell cancer and melanoma. In 90% of all squamous cell carcinoma and 50% of all basal cell carcinomas a mutation in the tumor suppressor p53 can be found [305]. In fact the most prominent mutation in p53 is a G to T transversion that could be a result of an oxidative modification of a guanine [306,307,308,309,310]. It is considered that a mutation in p53 leads to an inability of the cells to induce apoptosis, increasing the risk for a cancerous transformation [305,311]. Besides mutations in p53 mutations in Rb, adenomatous polyposis coli (APC) and patched (PTCH) also promote tumor formation [312,313]. ROS are not only involved in initiating tumor formation but also in its progression. It was found that tumor cells produce a high dosage of H_2_O_2_ that could be a key factor in tumor progression [306,314,315]. It was proposed that hydrogen peroxide is a second messenger that is capable of activating molecular switches. Examples of these switches are the redox sensitive Mitogen Activated Protein Kinases and other protein kinases: ERK1/2 (the extracellular signal-regulated kinases), JNK1/2/3 (the c-Jun NH2-terminal kinases), the p38 mitogen-activated kinases, P13K/Akt (phosphoinositide 3-kinase/serine-threonine kinase) and PKB (protein kinase B) [306,316,317,318]. All these pathways are known to regulate cell proliferation and migration. In general it can be stated that addition of ROS to cell cultures increases cell proliferation [319,320]. Other redox sensitive “switches” are transcription factors such as AP-1, NF-κB, NF-E2 related factor-1 (Nfr1), NF-E2 related factor-2 (Nrf2) and Egr1 (early growth response 1) [306,321,322] that modulate gene expression [298] which could contribute to cell proliferation and migration. In addition, ROS can initiate release of calcium from ER-localized stores. This release of calcium then activates PKC (protein kinase C) which is also involved in cell migration, apoptosis, proliferation and cytoskeletal reorganisation [298,323,324].

An irreversible alteration to a cell signaling pathway is the most common reason for the genesis of cancer, in the skin and elsewhere [325]. It was demonstrated that ROS are essential factors in melanomas. After melanocyte transformation the melanosomes are disorganized and, instead of scavenging, they promote ROS formation. These ROS were then shown to activate proto-oncogene pathways [326].

### 7.3. Senescence

One of the most powerful “tools” of a cell against a tumorgenic transformation is senescence [327,328,329]. An activation of the senescence program leads to an irreversible cell-cycle arrest in the G_1_-phase [330,331]. Keratinocytes and melanocytes, as well as fibroblast can become senescent [332]. Senescence associated beta-galactosidase, a marker of senescence, is found increasingly in aged tissues and aged skin [333]. In the skin. UV-radiation massively induces premature senescence and could in this way contribute to skin aging and photoaging [334]. The senescent cells stay alive, but start to change their behavior. They develop a very specific secretome, summarized as the “senescence-associated secretory phenotype”. In the dermis, senescent fibroblast also activate matrix metalloproteinases and express less matrix metalloproteinase inhibitors and extracellular matrix components like collagen [25,335,336]. Finally, senescent skin cells die by a mechanism that is either described as apoptosis or autophagic programmed cell death [337,338]. Senescence is initiated after such dramatic incidences as severe DNA damage, critical telomere shortening, oxidative stress and hyperactive oncogenic RAS [339,340,341,342]. In this context ROS can fulfil a dual role. At low concentration ROS can stimulate the proliferation of cells (discussed above), at high concentrations ROS seem to be involved in the induction of growth arrest of cells [343,344]. This replicative senescence phenotype can be either achieved by the p53/p21 or p16/Rb pathway [338]. Retinoblastoma protein (Rb) is responsible for the transition from the G_1_ to S phase. This transition is dependent on the phosphorylation state of Rb. Rb has the ability to bind to members of the transcription factor family E2F. Upon phosphorylation of this tumor supressor E2F transcription factors are released and the cell can pass from the G_1_ to the S phase. Rb can either be phosphorylated by the cycline dependent Kinase CDK4 or CDK6. The oxidative stress sensors in this regulatory pathway are the redox sensitive kinases ERK1/2 and p38. Their activation leads to an increased expression of p16. Overexpression of p16 was shown to induce senescence, whereas a p16 knock down was associated with an inhibition of RAS-mediated senescence. p16 is an inhibitor of several cycline dependent kinases. An activation of p16 via Erk1/2 and p38 leads to an inhibition of the cylcine-dependent kinases that are not able to phosphorylate Rb anymore. The tumor suppressor Rb is then in a complex with the E2F transcription factors, resulting in an inhibition of E2F target genes and a cell cycle arrest [343,345,346,347,348,349,350,351].

The second way to senescence leads through the tumor suppressor p53. p53 can either be activated by DNA damage or by oxidative stress. In healthy cells p53 has a short half-life. p53 is bound by Mdm2 that initiates its transport out of the nucleus resulting in its degradation. DNA damage leads to an activation of ATM (Ataxia telangiectasia mutated)/ATR (ataxia telangiectasia and Rad3-related) protein kinases via DNA damage response elements resulting in a p53 activation. The response to oxidative stress is mediated via p38 that directly phosphorylates p53. Phosphorylated p53 is no longer exported from the nucleus and initiates the transcription of genes involved in apoptosis and cell-cycle arrest. The main candidate for the cell cycle arrest is p21 that is a very potent cyclin-dependent kinase inhibitor acting on CDK2 and CDK4 [343,349,352,353,354,355]. In keratinocytes a special pathway exists to cope with the high UVB dosage. The UVB response of keratinocytes is tightly connected with the insulin-like growth factor-1 receptor. Activation of this receptor leads to ROS production and expression of p21 via p53 [334]. In more detail, senescence can be divided into acute senescence (like wound healing, development and injury repair) and chronic senescence (like aging) as described in van Deursen 2014 [343].

## 8. Oxidative Stress and Lipids

ROS molecules originating from different sources in the cell have the capacity to induce the lipid peroxidation process. This chain reaction starts with a reaction of a ROS molecule with polyunsaturated fatty acids. In a first step hydrogen atoms are removed from methyl groups of these lipids forming a lipid radical. In the next step a reaction with molecular oxygen takes place leading to a peroxyl radical. A reaction of this radical with another polyunsaturated fatty acid leads to a lipid peroxide and a new radical starting a chain reaction [356]. Oxidation of lipids is in discussion as being involved in human diseases such as atherosclerosis and cancer [357]. The most prominent end product of lipid peroxidation is 4-hydroxynonenal. The complex biology of 4-hydroxynonenal is treated in detail in the chapter by Jörg Schaur *et al.* elsewhere in this volume. Among other things the application of 4-hydroxynonenal to keratinocytes started a multitude of cellular responses such as an up-regulation of anti-oxidative enzymes (heme oxygenase-1 (HO-1), catalase, NADPH:quinone oxidoreductase (NQO1), and glutathione S-transferases), activation of several kinases such as Erk1/2, JNK and p38 and PI3 and a translocation of the transcription factor Nrf2 to the nucleus [358]. This is of special importance for the aging process because the level of 4-hydroxynonenal increases with aging leading to the cellular responses discussed above. In addition it was shown that in aged human fibroblasts derived from facial skin, 4-hydroxynonenal reacts with macromolecules, leading to the formation of hydroxynonenal modified proteins [359].

## 9. Conclusions

This chapter aims to give an overview of the role of oxidative stress in the general pathology and in the aging process of skin. In the epidermis a very pronounced phenotype is the disruption of the calcium gradient that results in a changed composition of the cornified envelope and changes in gene expression of other calcium dependent proteins of which only the S100 proteins shall be named. A reduced barrier function of aged skin is the major result. The aging process in the dermis is quite different, has other causes, and leads to other phenotypes. The process is driven by the activation of matrix metalloproteases, and the resulting degradation of the extracellular matrix components, especially collagens and elastic fibers. These ECM changes lead to the appearance of fine wrinkles. The aging process in the skin is driven by reactive oxygen species to an extent that is not attained in any other organ.

This review summarizes the most important contributors to ROS production that are common to all organs:

1. the mitochondrial electron transport chain
2. peroxisomal localized enzymes involved in the β-oxidation of fatty acids, the glyoxylate/dicarboxylate metabolism and the xanthine oxidase, involved in purine catabolism
3. the endoplasmic reticulum, localized enzymes, protein disulfide isomerase, and endoplasmic reticulum oxidoreductin-1 ERO1 as well as members of the cytochrome P450 family
4. the enzymes cyclooxygenase and lipoxygenase involved in the arachidonic acid metabolism, the Fenton reaction and the membrane localized NADPH oxidase family

Because the skin is at the interface between the exterior and the interior, external factors also contribute to ROS production in the skin. Besides xenobiotics and pollutants the main factor amplifying the photoaging process in the skin is UV irradiation.

Typical defense mechanisms against this high ROS burden are enzymes such as superoxide dismutases, catalase, peroxiredoxins, GSH peroxidases, as well as non-enzymatic components such as L-ascorbate, α-tocopherol, beta-carotene, uric acid, CoQ10, and the whole glutathione system. The skin is equipped with several of these enzymes, including enzymatic and non-enzymatic antioxidants. In addition there are water-soluble antioxidants like glucose, pyruvate, and bilirubin as well as lipid-soluble antioxidants like α-tocopherol, ubiquinol-10, lycopene, and β–carotene present. Interestingly the predominant antioxidants found in skin cells are nonenzymatic. This includes L-ascorbic acid, GSH, and uric acid [360].

In contrast to the dermis the epidermis has higher levels of catalase, glutathione peroxidase, glutathione reductase, and ascorbic acid. In addition the *stratum corneum* is equipped with Vitamins C and E as well as uric acid [361,362,363]. Another defense mechanism against ROS is the SPRR protein family, which is also highly present in the *stratum corneum*. These proteins are able to efficiently quench ROS by forming intramolecular disulfide bonds.

The highest concentrations of antioxidant enzymes (and the SPRR proteins) can be found in the outermost layer of the epidermis. This is due to the fact that the O_2_ partial pressure is higher at the surface presenting another source for ROS. Unquenched ROS molecules lead to the formation of protein carbonyls, advanced glycation end products, lipofuscins, start the process of lipid peroxidation and lead to pronounced DNA damage (especially 8-oxo-2'deoxyguanosine).
